# Improving radiation therapy for non-small cell lung cancer: molecular imaging and a team-based approach

**DOI:** 10.2349/biij.3.1.e3

**Published:** 2007-01-01

**Authors:** SJ Everitt, M Mac Manus

**Affiliations:** 1 Radiation Therapy Services, Peter MacCallum Cancer Centre, Melbourne, Australia; 2 Department of Radiation Oncology, Peter MacCallum Cancer Centre, Melbourne, Australia

**Keywords:** Positron emission tomography, radiation therapy, 3DCRT, non small cell lung cancer

## Abstract

The successful integration of molecular imaging and radiation therapy has been shown to significantly impact the management of patients with non-small cell lung cancer (NSCLC). The collaboration of multidisciplinary team members, including radiation oncologists, radiation therapists, nuclear medicine physicians and physicists, has enabled PET/CT to be utilised for routine use throughout the radiotherapy treatment trajectory. Applications include disease diagnosis and staging, target volume definition for radiation therapy and monitoring tumour response to treatment. Not only has the adoption of this technology demonstrated benefits for our current patients, it is also opening doors for significant research in the future.

The safe and accurate delivery of radiation therapy (RT) to patients with cancer has always demanded a team approach. For much of the history of RT, treatments could be planned and delivered by a small team that included a physician and a radiation therapist together with the engineering and medical physics staff required to ensure that treatment machines gave the output that was prescribed. In recent years, as treatments have become enormously more complex and somewhat more effective, the membership of the RT team has increased. The planning and delivery of RT frequently requires contributions from radiation oncologists, radiation therapists (also known as therapy radiographers in many countries), medical physicists and dosimetrists, together with nuclear medicine and diagnostic imaging specialists. In the past, many of these specialist disciplines have tended to operate in autonomous environments. However, with the burgeoning complexity of RT planning and delivery, these disciplines are increasingly integrating to combine their expertise for the benefit of patients treated with RT.

One of the most striking recent examples of change is the increasingly close involvement of nuclear medicine in patient selection and RT treatment planning. Positron emission tomography (PET), primarily using 2-[^18^F] fluoro-2-deoxy-D-glucose (FDG) as the radiopharmaceutical, has increasingly been employed in the diagnosis and staging of non-small cell lung cancer (NSCLC) and other common cancers. In NSCLC, many studies have been published showing that PET has a significant impact on the selection of patients for curative therapy, most commonly surgery, by identifying candidates without gross systemic metastatic disease and without intrathoracic disease too extensive for an attempt at cure. PET scanning was first employed at the Peter MacCallum Cancer Centre (Peter Mac) as a staging tool for patients with NSCLC a decade ago. At that time, we commenced the first large study of the use of PET staging for NSCLC patients who were candidates not for surgery but for radical RT. In this prospective study PET staging was conducted for 153 patients considered eligible for radical RT based on the results of conventional staging investigations. Results of this study published by Mac Manus *et al* revealed that 46/153 (30%) patients were deemed ineligible for radical RT following PET because of detection of distant metastatic disease or intrathoracic disease too extensive for radical radiation [1]. Mah *et al* reported similar findings, stating that where PET data were incorporated into the disease staging process the treatment intent was changed from radical to palliative for 7 out of 30 patients (23%) [2]. Hicks *et al* supported the work of other researchers, reporting that in such cases where PET indicated a poor prognosis, patients were spared from the lengthy duration and unwarranted morbidity of futile aggressive treatment [3]. In addition to ensuring that only those patients who are most likely to benefit from curative therapy were treated intensively, the significant costs and resources associated with such radical treatment were also avoided in these cases. These results have led to the routine incorporation of molecular imaging into the RT planning process at our centre.

In recent years there have been many attempts to improve outcomes for patients with unresectable but still potentially curable NSCLC through altered dose and fractionation regimens and by prescribing radiation in combination with a wide range of chemotherapeutic agents. The most successful of these efforts have been the use of continuous hyper-fractionated radiotherapy (CHART) [4] and platinum based chemotherapy combined with RT [5], both of which give superior survival to conventional RT alone. The importance of tumour imaging for RT target delineation and dosimetry is becoming increasingly recognised and is an area of intense interest at present. Even the most effectively fractionated or highly chemo-sensitised RT will have a low chance for success if the tumour is not effectively contained within the high dose RT volume. Until the late 1990’s, target volumes for RT planning were based solely on diagnostic CT scan data, a practice that continues in many centres today. Protocols for tumour delineation routinely documented that the gross tumour volume (GTV) included the primary disease and ipsilateral hilar and mediastinal lymph nodes, which were electively irradiated irrespective of their radiologic appearance. Since tumour volume delineation was based on CT alone, all lymph nodes thought to be involved (>1cm short axis diameter) were also encompassed in the volume. In centres where elective nodal irradiation is not routine, a 1.5–2.0cm margin is typically applied to the GTV, and an additional margin is often applied to allow for motion of the tumour with the respiratory cycle, the sum of these, thereby generating the planning target volume (PTV). The PTV is the volume that must be treated as uniformly as possible to the prescribed radiation dose and critical tissues outside this volume should be treated to as low a dose of radiation as possible.

With the advent of PET imaging, the process of defining RT target volumes is changing. Many studies have reported the potential advantages of PET-assisted target volume definition in NSCLC [1,2,6-15]. These studies suggest that the benefits of PET in staging this disease, particularly through more reliable identification of tumour bearing lymph nodes, also translate into superior target definition. The superior accuracy of PET over CT in staging mediastinal lymph nodes has been demonstrated by a large number of prospective studies [16-21]. In a comprehensive meta-analysis Toloza *et al* reviewed 18 studies that used PET and 20 that used CT for staging mediastinal disease [16]. They demonstrated that the accuracy of CT scanning for mediastinal staging had not improved over the past decade, despite improvements in CT scan resolution [16]. Of the 3438 patients examined, the pooled sensitivity of CT scanning was 0.57 (95% CI, 0.49 to 0.66), and the pooled specificity was 0.82 (95% CI, 0.77 to 0.86). Another meta-analysis of 42 CT studies performed between 1980 and 1988 reported sensitivities of 79% and specificities of 78% [22]. The superiority of PET is highlighted in the meta-analysis by Toloza *et al*, where sensitivity and specificity for mediastinal staging were reported as 84% (95% CI, 0.78 to 0.89), and 89% (95% CI, 0.83 to 0.93), respectively. Other authors not included in the analysis by Toloza *et al* have also reported similar results [17,18, 23]. Overall, these authors have demonstrated increased evidence and confidence in the ability of PET to detect tumour in normal sized lymph nodes and also to exclude tumour in abnormally enlarged nodes. As a result of these findings, the radiotherapeutic management of patients with mediastinal involvement can reasonably be altered by taking PET information into account [18].

Another significant advantage of PET in target volume delineation is its ability to differentiate tumour from atelectatic lung with greater accuracy than other imaging modalities [6,7,9,12]. This is particularly difficult to achieve using the morphologic information given by CT [11]. Without PET information, target volumes may incorporate unnecessarily large volumes of disease free collapsed or consolidated lung. Conversely, 3DCRT target volumes based on PET/CT information focus radiation on metabolically active disease, thereby sparing adjacent normal lung tissue from unnecessary dose and reducing the potential for radiation pneumonitis.

At our institution we began to incorporate PET information into the process of tumour volume definition for patients planned to receive RT for NSCLC in 1996. Radiation oncologists incorporated PET data into the RT treatment planning process simply by visually estimating the location and extent of PET positive structures on PET hard copies in relation to anatomical landmarks on planning CT scans. The impact of this method was assessed for 102 eligible patients at our centre [1]. Overall, 41/102 (40%) patients required changes to their RT plan to ensure appropriate treatment of tumour detected by PET. In 22/102 (22%) cases PET led to a significant increase in the target volume because of inclusion of structures previously considered not involved by tumour. In 16/102 (16%) cases the target volume was significantly reduced, where PET demonstrated areas of lung collapse or consolidation and/or enlarged lymph nodes with low 18F-FDG uptake that were excluded from the treatment volume. In addition to this, primary tumours seen on PET were not identified on CT in 3/102 (3%) patients.

This was an ad-hoc, low technology method that did not fully utilise the three-dimensional information from PET. At the time we had no means of incorporating PET information directly into the treatment planning software. As previously published, one of our physicists overcame this barrier by writing software that allowed importation and co-registration of separately acquired PET and CT images [24]. This technique was investigated for 10 consecutive patients with NSCLC. The method was robust and practical and we saw similar changes in PET/CT plans compared to CT-alone plans to those that were observed in our earlier studies performed without co-registration.

Other studies have demonstrated similar findings to our own [2,6,8,9,12-14]. Bradley *et al* reported that 14/24 patients (58%) planned for definitive RT had significant alterations in the GTV and PTV, attributable to the detection by PET of additional nodal (n=10) or primary disease (n=1) or to the demarcation of gross tumour within atelectatic lung (n=3) [6]. Erdi *et al* reported that the PTV was increased in 7/11 (64%) patients studied, to incorporate additional regional nodal disease detected with PET [8]. In a retrospective study, Nestle *et al* reported that the incorporation of overall PET findings altered the shape of the radiation portals in 12/34 (35%) patients [12]. Similarly, Kiffer *et al* reported the use of PET images for planning would have altered the RT portals in 7/15 patients (47%) [13]. In all studies, the inclusion of PET has had a significant impact on target volume definition in a substantial proportion of patients (approximately 30–60%), and in those cases PET has influenced the design of the PTV and consequently the design of RT dosimetry to ensure optimal coverage of the tumour. Each of these changes could be expected to lead to more accurate delineation of target volumes for 3DCRT. In turn, improvements in tumour coverage may have facilitated improved patient outcomes through minimised risks of excluding gross tumour and avoidance of unnecessarily irradiating surrounding normal tissues, although this would be exceedingly difficult to prove.

In 2001, the Centre for Molecular Imaging at Peter Mac acquired an integrated PET-CT scanner, providing true fused images for RT planning. Our previous co-registration method became obsolete at a stroke. Because PET and CT data are acquired at a single session potential inaccuracies associated with separate acquisitions were eliminated, including patient position reproducibility, different breathing patterns and errors associated with fiducial marker co-registration and image registration. Both CT and PET data are readily visualised simultaneously on the RT planning computer for target volume delineation. This system is now routinely used for all patients treated with radical RT for NSCLC and oesophageal cancer at our centre. PET information is also commonly used to assist with RT target definition in cervix and head and neck cancers, paediatric cancers and lymphomas.

Successfully integrating this technology into routine practice has relied upon continuous and effective communication between disciplines in molecular imaging and RT, including physicians, radiation therapists, nuclear medicine technologists and medical physicists. The nuclear medicine physician plays a key role in assisting the radiation oncologist to accurately contour gross tumour. Radiation oncologists generally have little training in PET and without expert support may not use PET information effectively. As a team we acknowledged the potential pitfalls and sources of error involved in this process and invested considerable effort to ensure reproducibility of scanning conditions and consistency of PET/CT image display on RT planning computers. Because radiation oncologists undertake target volume delineation in close consultation with their diagnostic imaging colleagues, a true multi-disciplinary assessment occurs. We therefore believe that the highest quality information available to us is used for target volume determination.

A key goal of research in radiation oncology is to maximise the therapeutic ratio. The addition of PET to CT for defining target volumes for RT has the potential to help achieve this goal by targeting the tumour accurately and sparing normal structures previously thought to contain tumour. We are currently conducting a prospective study that will recruit 50 patients who go on to receive radical RT for NSCLC after PET staging. A similar study is commencing in the USA under the auspices of the Radiation Therapy Oncology Group (RTOG). Each of these studies will compare dosimetry based on tumours defined with PET/CT compared to volumes derived using CT alone. In time, we hope that valuable information relating to tumour control, normal tissue toxicity and patient survival will validate the impact of PET on overall patient outcomes.

Apart from initial target volume definition, there remains great potential to further improve outcomes for patients with NSCLC. Preliminary research has explored the value of integrating PET data into during-treatment and post-treatment tumour assessment. A recent Peter Mac study by MacManus *et al* investigated patterns of metabolic tumour response and disease progression for 88 patients after PET information was used together with CT to stage and plan radical RT [3]. 73/88 (83%) patients received concurrent platinum-based radical chemo/RT and 15/88 (17%) received radical RT alone. A restaging PET scan, performed to investigate patterns of metabolic tumour response, was conducted at a median time of 70 days after treatment. The scan results demonstrated that the tumour was stable in 72/88 (81%) patients, including 40/88 (45%) who had attained a complete metabolic response. However, by the final follow-up at four years 70/88 (80%) patients demonstrated progressive disease with disease relapsing locally in 62/88 (71%), either alone or in combination with distant metastasis. Only 17/88 (19%) patients survived for four years. Of all the patients who attained a complete metabolic response half eventually had local failure. The very high rate of local progression after radical RT confirms yet again that a prescribed radiation dose 60Gy is inadequate to control more than a low percentage of lung cancers, even when combined with concurrent chemotherapy. Nevertheless, the high rate of isolated loco-regional recurrence suggests that intensification of local therapy could potentially improve outcomes in future clinical trials.

In conclusion, we believe that the combination of advanced imaging with advanced RT planning is an excellent example of how teamwork and a true multidisciplinary approach can help us harness new technology for the future benefit of our patients. New avenues for research are opening up that suggest that the future potential of this approach is immense.
